# Bispecific antibodies with Fab-arms featuring exchanged antigen-binding constant domains

**DOI:** 10.1016/j.bbrep.2021.100959

**Published:** 2021-02-27

**Authors:** Filippo Benedetti, Florian Stracke, Gerhard Stadlmayr, Katharina Stadlbauer, Florian Rüker, Gordana Wozniak-Knopp

**Affiliations:** CD Laboratory for Innovative Immunotherapeutics, Institute of Molecular Biotechnology, Department of Biotechnology, University of Natural Resources and Life Sciences (BOKU), Vienna, Muthgasse 18, 1190, Vienna, Austria

**Keywords:** Bispecific antibody, Fab constant domain exchange, Domain-exchanged antibody, “Knobs-into-holes” heterodimerization, Her2 internalization, Ab, antibody, BLI, biolayer interferometry, BSA, bovine serum albumin, CDR, complementarity determining region, DSC, differential scanning calorimetry, EC_50_, half-maximal effective concentration, Fab, fragment antigen binding, FBS, fetal bovine serum, Fc, fragment crystallizable, Fcab, Fc with antigen binding properties, FITC, fluorescein isothiocyanate, HPLC-SEC, high pressure liquid chromatography-size exclusion chromatography, IgG, immunoglobulin G, LC-ESI-MS, liquid chromatography-electrospray ionization-mass spectrometry, PBS, phosphate buffered saline, PE, phycoerythrin, PEI, polyethylenimine, PNGase F, Peptide:N-glycosidase F, RMSD, root mean square deviation, *Tm*, melting temperature, TRA, trastuzumab, VEGF, vascular endothelial growth factor

## Abstract

Monoclonal antibodies can acquire the property of engagement of a second antigen via fusion methods or modification of their CDR loops, but also by modification of their constant domains, such as in the mAb^2^ format where a set of mutated amino acid residues in the C_H_3 domains enables a high-affinity specific interaction with the second antigen. We tested the possibility of introducing multiple binding sites for the second antigen by replacing the Fab C_H_1/C_L_ domain pair with a pair of antigen-binding C_H_3 domains in a model scaffold with trastuzumab variable domains and VEGF-binding C_H_3 domains. Such bispecific molecules were produced in a “Fab-like” format and in a full-length antibody format. Novel constructs were of expected molecular composition using mass spectrometry. They were expressed at a high level in standard laboratory conditions, purified as monomers with Protein A and gel filtration and were of high thermostability. Their high-affinity binding to both target antigens was retained. Finally, the Her2/VEGF binding domain-exchanged bispecific antibody was able to mediate a potentiated surface Her2-internalization effect on the Her2-overexpressing cell line SK-BR-3 due to improved level of cross-linking with the endogenously secreted cytokine. To conclude, bispecific antibodies with Fabs featuring exchanged antigen-binding C_H_3 domains offer an alternative solution in positioning and valency of antigen binding sites.

## Highlights

• Fab constant domains can be efficiently exchanged for antigen-binding C_H_3 domains.
• Such mutagenesis results in bispecific antibodies with correct chain pairing.
• Domain-exchanged bispecific Fab- and IgG-like formats are of favorable biophysical properties.
• Resulting bispecific antibodies show high-affinity binding to both target antigens.

## Graphical abstract

**Figure:**
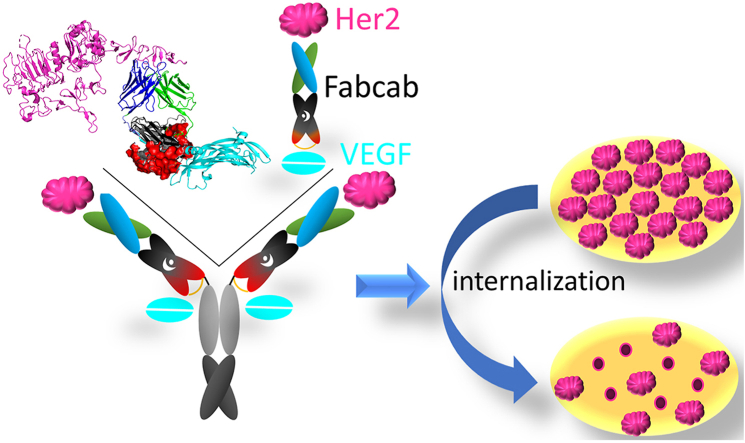

## 1 Introduction

In the past two decades, bispecific antibodies have developed into one of the most promising classes of antibody-based therapeutic reagents. The intensity of research invested in the innovative designs, manufacturability and beneficial novel biological functions of these molecules has resulted in two clinically approved therapeutics and further seven candidates in late-stage clinical studies at the end of 2019 [1]. Their unique property of joining two antigen specificities in a single molecule enabled novel therapeutic approaches such as mobilization of T cells for specific tumor targeting, bridging of two enzymes to compensate for the activity of the missing catalytic cascade factor, or combating tumor extravasation and immune escape by blocking two cytokines mediating redundant metabolic pathways [2].

Since the initial attempts to produce bispecific antibodies with reduction and re-oxidation of two different antibody fragments [3,4], protein engineering methods have been applied extensively for the design of well-expressed and highly soluble bispecific agents, resulting in over 100 different formats available today [2]. These include symmetric as well as heterodimeric molecules that are either antibody fragments, exhibit a native IgG-like architecture, or are fusion proteins. The large variety of formats implies high variability in spatial positioning of the different antigen-binding sites, and even more importantly, in the stoichiometry of binding of antigen molecules, which can be chosen at will depending on the foreseen biological setting. The importance of these two factors has been documented with many examples of clinical significance: closer positioning of binding sites in a bispecific antibody fragment specific for target tumor cells and effector cells results in more potent specific cell killing [5], monovalent engagement of CD3 on the effector cells by bispecific antibodies increases safety of the therapeutic agent due to diminished probability of cytokine release syndrome [6], and agonistic anti-OX40 antibodies that require multivalent or multi-paratopic binding to OX40 to achieve high-order receptor clustering that enables full activation of T-cell co-stimulatory pathways [7].

One novel bispecific antibody format that has already entered clinical trials is mAb^2^ [[8], [9], [10]], a monoclonal antibody with the binding site for the second antigen introduced into the C-terminal structural loops of the C_H_3 domains of the Fc fragment, rendering this part of the molecule an antigen-binding Fc fragment (Fcab) [11]. Homodimeric bispecific mAb^2^ binds two antigen molecules with the Fab arms, and either one or two molecules of the second antigen can be captured with the binding site formed with the mutated amino acid residues in the C_H_3 domains. As the specifically binding mutated C_H_3 domains are typically sourced by phage and yeast display library screening [[11], [12], [13]], avidity effects upon the selection with multivalent antigens mostly favor bivalent binders [14,15]. In certain cases, heterodimerization of an antigen-binding Fc fragment and exchange of one of the antigen-binding domains for a wild-type C_H_3 can lead to only a small decrease in binding affinity to a dimeric antigen [15].

Regarding the potential value of introducing multiple antigen-binding sites in a mAb^2^ molecule, we attempted to harvest the finding that constant domains of the Fab fragment can be exchanged for the C_H_3 domains, and such molecules can readily be produced in mammalian expression system and exhibit favorable biophysical properties [16]. We have chosen the high-affinity anti-VEGF Fcab clone CT6 that binds to the cognate antigen in 1:1 stoichiometry with 4 nM-affinity *via* mutations located in all 3 C-terminal structural loops and the C-terminus of the C_H_3 domain [17], and produced the derived antigen-binding domain-exchanged Fab-like fragment (named FabCab for *Fab* with *a*ntigen-*b*inding *c*onstant domains), as well as the bispecific full-length domain-exchanged antibody, with variable domains of trastuzumab, the Her2-specific blockbuster antibody [18,19]. The biophysical properties of the novel molecules, such as monomeric status, thermostability, and the accurateness of heterodimer formation were examined, as well as their binding to both antigens and the ability of simultaneous bispecific binding. We further anticipated that the bispecific antibody constructed in this way could enable efficient internalization of surface Her2 upon cross-linking with dimeric VEGF, and exhibit a higher efficiency than the Her2/VEGF-specific mAb^2^, composed of trastuzumab variable domains and the CT6 Fcab, due to multiple binding sites for this cytokine.

## 2 Materials and methods

### 2.1 Production of the domain-exchanged antibodies

#### 2.1.1 Design and construction

For the design of the Her2/VEGF-specific domain exchange antibody, we were able to apply many principles validated for the construction of such molecules with wild-type C_H_3 domains: the junctions between heavy and light chains were introduced as described previously [16], and the influence of the scaffold mutation F404Y (EU numbering scheme) [20] that was reported to enhance the solubility and the thermostability of the domain-exchanged antibody construct [16] was first tested in domain-exchanged Fab-like proteins with a single antigen-binding C_H_3 domain and introduced into the full-length IgG-like constructs upon obtaining the evidence of its beneficial effect. The C-terminal residues -GEC required to form the interchain disulfide bond were introduced after the residue W445 as this one was shown to be indispensable for thermal stability of the construct, being involved into a CT6-particular network of aromatic rings and contributing to π-π stacking, and also to form van der Waals’ interactions with VEGF [15].

The Her2-VEGF specific mAb^2^ was constructed by replacing the C_H_3 domain of trastuzumab sequence cloned in the pTT5 vector (CNRC) with the C_H_3-domain sequence of the CT6 Fcab (amino acid sequences of all constructs are listed in Supplementary Table 1). Site-directed mutagenesis kit (Agilent) was used to introduce the heterodimerization mutations “Knob” (T366Y) and “Hole” (Y407T), as well as the F404Y mutation into the CT6 sequence. Subsequently, mutated C_H_3-domains were introduced into the pTT5-based vectors: the “Knob”-variant (T366Y) was cloned in frame with TRA-V_H_-encoding sequence and the “Hole”-variant (Y407T) was fused with the TRA-V_L_-encoding sequence, to minimize the likelihood of formation and purification of homodimeric “Hole-Hole” species.

**Fig. 1:**
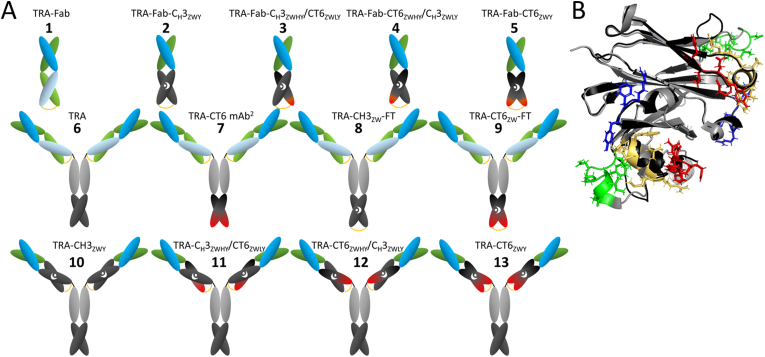
(A) Schematic of domain-exchanged and control constructs: V_L_: green, C_Κ_: light green, V_H_: blue, C_H_1: light blue, C_H_2: gray, C_H_3: black, C_H_3 with antigen-binding properties: gradient of black to red. Heterodimerization is indicated with white dot and crescent; (B) superimposition of wild-type C_H_3 domain (black) (PDB: 5JII) and CT6-C_H_3 domain (PDB: 5K65) (gray). Mutated residues are depicted with their side chains in blue (AB loop), green (CD loop), yellow (EF loop) and red (C-terminus). Figure was prepared using PyMOL (Schrödinger, Inc.). (For interpretation of the references to color in this figure legend, the reader is referred to the Web version of this article.)

As an alternative method of C_H_3-domains heterodimerization, a set of mutations that enables expression of heterodimeric antibodies with wild-type-like thermostability properties (ZW1-heterodimerization motif [21]), was tested in the mutated C_H_3-domain constructs. The chains TRA-V_L_-C_H_3_ZWL_ and TRA-V_H_-C_H_3_ZWH_ were constructed as described before [16] and TRA-V_L_-CT6_ZWL_ and TRA-V_H_-CT6_ZWH_ chains were constructed using Site-directed mutagenesis kit (Agilent), using TRA-V_L_-C_H_3_ZWL_ and TRA-V_H_-C_H_3_ZWH_ sequences as templates and oligonucleotides to introduce the mutations characteristic for the C_H_3 domain of the CT6 Fcab clone. Monovalent bispecific constructs (Constructs 2–5 in Fig. 1A) were cloned also as variants containing the F404Y mutation (TRA-V_L_-CT6_ZWLY_ and TRA-V_H_-CT6_ZWHY_). These were then used for construction of full-length domain-exchanged antibodies (Constructs 10–13 in Fig. 1A). To produce the full-length IgG-like antibodies with trastuzumab variable domains and C-terminally positioned heterodimerized wild-type C_H_3 or CT6 domains (named TRA-C_H_3_ZW_-FT and TRA-CT6_ZW_-FT for *f*ull length-*t*erminal; Constructs 8 and 9 in Fig. 1A)), the sequences encoding C_H_3_ZWH_ and CT6_ZWH_ chains were used to replace the wild-type C_H_3 domain of the pTT5-cloned trastuzumab sequence.

#### 2.1.2 Expression and purification

Antibody chains cloned into pTT5-based expression vectors were used for PEI-mediated transient transfection of HEK293-6E cells (CNRC) according to manufacturers' instructions, in 1:1 mass ratio for mAb^2^, Fab-like constructs and domain-exchanged antibodies encoding chains and 2:1:1 mass ratio of V_L_-C_L_: V_H_-C_H_1-C_H_2-C_H_3_ZWL_ (or CT6_ZWL_): V_H_-C_H_1-C_H_2-C_H_3_ZWH_ (or CT6_ZWH_) to produce Constructs 8 and 9. Two days post transfection, cells were fed tryptone TN-20 to a final concentration of 0.5% and supernatant was harvested 5 days post transfection with centrifugation at 1000*g*, 20 min at 4 °C and immediately used for purification with Protein A. The VEGF isomer 109 with N75Q mutation (numbering as in PDB: 1VPF [22]) to obliterate the N-linked glycosylation site was produced in the same expression system and purified with Ni-NTA chelating affinity chromatography as described before [17]. Biotinylation was performed with NHS-LC-LC-biotin (Thermo Fisher Scientific) at a ratio of 1:1.5 protein to biotin, exactly according to manufacturer's instructions.

FabCab and the domain-exchanged antibody with CT6-mutations on both heavy and light chains were produced in ExpiCHO system (Thermo Fisher Scientific) according to the instructions outlined in the MaxTitre protocol by the manufacturer. Supernatants were harvested 12–14 days post transfection with centrifugation at 1000*g* and further clarified with a centrifugation step at 3000*g*, 20 min at 4 °C. Clarified supernatants were buffered with 0.1 M Na-phosphate buffer, pH 7.0, and filtered through a 0.45 μm filter. The samples were then passed over Protein A HP column (GE Healthcare), equilibrated with the same buffer. The column was washed with 20 column volumes of 0.1 M Na-phosphate buffer, pH 7.0, and bound proteins were eluted with 0.1 M glycine, pH 3.5. Fractions were neutralized immediately and dialyzed against a 100-fold volume of PBS overnight at 4 °C. The proteins were stored at −80 °C until use.

### 2.2 Biophysical characterization

#### 2.2.1 SEC-HPLC

Shimadzu LC-20A Prominence system equipped with a diode array detector was used to perform SEC-HPLC with a Superdex 200 Increase 10/300 GL column (GE Healthcare). The mobile phase buffer used was PBS with 200 mM NaCl. Chromatography was conducted with a constant flow rate of 0.75 mL/min. A total of 20 μg protein at about 1 mg/mL were loaded on the column for analysis. Column calibration was performed with a set of molecular weight standards ranging from 1.3 to 670 kDa (Bio-Rad).

#### 2.2.2 DSC

DSC experiments were performed using an automated MicroCal PEAQ-DSC Automated system (Malvern Panalytical), using 5 μM protein solution, diluted in PBS at pH 7.5. The heating was performed from 20 °C to 100 °C at a rate of 1 °C/min. Protein solution was then cooled *in situ* and an identical thermal scan was run to obtain the baseline for subtraction from the first scan. All measurements were taken in duplicates. Fitting was performed with Origin 7.0 for DSC software using the non-2-state transition mechanism.

#### 2.2.3 Mass spectrometry

20 μL of glycosylated or PNGaseF digested sample (β = 0.30 mg/mL) was analyzed using a Dionex Ultimate 3000 LC-ESI-MS system directly linked to a QTOF instrument (maXis 4G ETD, Bruker) equipped with the standard ESI source in the positive ion, MS mode (range: 750–5000 Da) mode. Instrument calibration was performed using ESI calibration mixture (Agilent). For separation of the proteins a Thermo ProSwift™ RP-4H Analytical separation column (250 * 0.200 mm) was used. A gradient from 20 to 80% acetonitrile in 0.05% trifluoroacetic acid at a flow rate of 8 μL/min was applied in 30 min gradient time. Deconvolution of summed spectra was done using the MaxEnt algorithm in Data Analysis 4.0 (Bruker).

### 2.3 Antigen-binding properties

#### 2.3.1 Cell surface binding and bispecific binding

SK-BR-3 cells (ATCC®-HTB30™) were cultured in DMEM with 10% FBS and penicillin-streptomycin in humidified atmosphere at 37 °C under 5% CO_2_. Cells were harvested with Biotase detachment reagent (Biochrom), resuspended to a density of 1x10^6^ cells/mL and blocked with 2% BSA-PBS for 30 min on ice. 100-μL-aliquots were distributed into the wells of a 96-well-plate. After centrifugation for 5 min at 300*g* and 4 °C, the cells were stained with 3-fold serial dilution of test antibodies in 2% BSA-PBS for 30 min on ice. A 1:1000 dilution of anti-human gamma chain-PE conjugate (Sigma-Aldrich) in 2% BSA-PBS was used to detect test protein binding. One exception here was Construct 5 (TRA-Fab-CT6_ZWY_), where we could not measure any specific reactivity with the mentioned conjugate, and hence employed the Zenon™ Alexa Fluor™ 647 labelling reagent (Thermo Fisher Scientific) at a dilution of 1:1000 instead. For control, we also evaluated this secondary reagent in combination with TRA-Fab-C_H_3_ZWY_ and determined the same EC_50_ as before. To determine the ability of bispecific binding, the incubation of test antibodies was followed by incubation with 10 μg/mL biotinylated VEGF in 2% BSA-PBS for 30 min on ice, and bound antigen was detected after the incubation with 1:1000 dilution of streptavidin-Alexa Fluor™ 647 (Thermo Fisher Scientific) in 2% BSA-PBS for 30 min on ice. Mean fluorescence of the cell population was determined with Guava® easyCyte™ flow cytometer (Luminex). EC_50_ values were derived after fitting data in 4-parameter-equation using Sigma Plot 13.0 software.

#### 2.3.2 Kinetic analysis of VEGF binding

The kinetic parameters of VEGF binding at 25 °C were determined using biolayer interferometry (BLI) with Octet RED96e system (ForteBio, Molecular Devices). Streptavidin tips, equilibrated in assay buffer (PBS with Kinetic Buffer) (ForteBio, Molecular Devices) were loaded with 10 μg/mL biotinylated VEGF for 300 s with agitation at 1000 rpm. After the recoding of second baseline, antibody fragments and antibodies in 2-fold serial dilutions starting from 125 nM were allowed to bind for 600 s and the tips were then immersed into assay buffer for 900 s for dissociation. Sensorgram curves resulting from antibody dilutions binding to non-coated tips and VEGF-coated tip immersed into assay buffer in the association and dissociation step were subtracted as background before fitting the response curves and determining kinetic parameters of binding using ForteBio Analysis software version 11.0. In the reverse outlay, anti-human Fc tips (ForteBio, Molecular Devices) were loaded with 50 μg/mL full-length IgG-like constructs, and the interaction with VEGF in 2-fold serial dilutions starting from 500 nM was measured with the same assay parameters as described above.

#### 2.3.3 Her2 internalization

SK-BR-3 cells were seeded into 6-well plates at a density of 300000 cells/well and allowed to attach overnight. Then they were treated for 48 h with 100 nM solution of the antibodies, and the controls included untreated cells as well as a mixture of trastuzumab and CT6 Fcab in equimolar or 1:2 ratio. Treated cells were then harvested using cell dissociation reagent and resuspended in cell culture medium containing 10% FBS. Cells were blocked with 2% BSA-PBS for 30 min on ice and stained for surface levels of Her2 with an anti-Her2 antibody 9G6 (Santa Cruz), recognizing a distinct epitope from TRA, at 1 μg/mL in 2% BSA-PBS for 30 min on ice. Its binding was detected with an anti-mouse-(Fab)’_2_ – FITC conjugate (Sigma-Aldrich), used at 1:200 dilution in 2% BSA-PBS for 30 min on ice. Fluorescence levels were recorded with Guava® easyCyte™ flow cytometry instrument and expressed as % fluorescence of untreated cells. Results of two independent experiments were evaluated.

## 3 Results

In the present work, we set out to explore the feasibility of the concept of producing bispecific antibodies by replacing the constant domains in Fab-fragments with antigen-binding C_H_3 domains (scheme of all constructs in Fig. 1A and their list in Supplementary Table 2). The initial idea was to apply the engineering principles established for antibody fragments and antibodies with constant domains of the Fab fragments exchanged for wild-type C_H_3 domains [16] as upon alignment of the C_H_3 domains of the wild-type Fc (PDB: 5JII) and CT6 (PDB: 5K65) an RMSD of only 0.384 Å was found (Fig. 1B).

The chosen scaffold antibody was trastuzumab and the source of antigen-binding C_H_3 domains was the CT6 Fcab, an anti-VEGF clone matured previously to high affinity. Both molecules exhibit high expression levels in HEK293-6E system (300 mg/L and 50 mg/L) and high solubility over 5 mg/mL. To produce a bispecific FabCab fragment and a corresponding full-length IgG-like domain-exchanged antibody, first the simple “Knobs-into-Holes” heterodimerization strategy [23], including only a single mutated amino acid residue per chain (T366Y/Y407T), was applied. However, the introduction of even only one chain containing C_H_3 domains with mutations that could mediate antigen-binding led to expression of molecules with aberrant HPLC profiles (Supplementary Fig. 1), and we therefore discontinued their further analysis. On the contrary, the FabCab fragments with one chain with the antigen-binding activity as well as their full-length IgG counterparts could be expressed as monomeric molecules when the heterodimerization was achieved with ZW1-heterodimerization motif (Supplementary Fig. 1). The yields after Protein A affinity chromatography of the FabCab proteins were about 50 mg/L for Construct 3 and about 20 mg/L for Construct 4, and about 50 mg/L for the corresponding domain-exchanged full-length IgGs (Construct 11 and 12). The expression level of Fab-like fragment (Construct 5) as well as the domain-exchanged full-length IgG with both heterodimerized antigen-binding C_H_3 domains (Construct 13) in HEK293-6E cells was not sufficient for characterization, but 50 mg and 60 mg of soluble protein could be purified from 1 L supernatant of ExpiCHO cells, respectively.

**Fig. 2:**
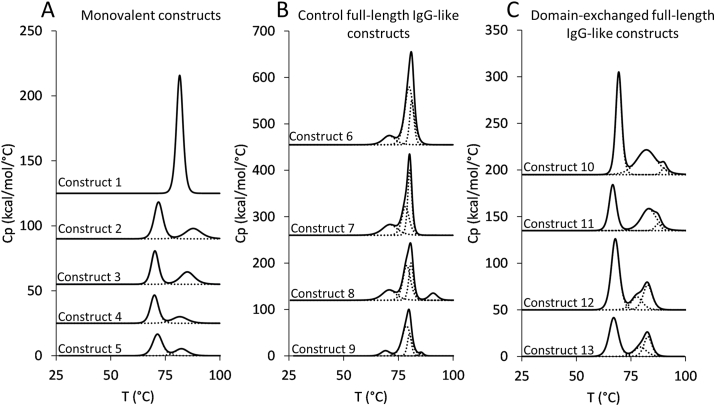
DSC profiles of (A) Fab-like domain-exchanged and Fab control constructs; (B) control full-length IgG-like constructs including with heterodimerized C-terminally positioned C_H_3 domains; (C) domain-exchanged full-length IgG-like antibodies.

The constructs were tested for their thermostability with DSC analysis (Fig. 2). For the monovalent fragments the thermogram could be deconvoluted with two transitions, the first corresponding to the unfolding of Fab variable domains around 71 °C and the second resulting from denaturation of the exchanged C_H_3 domains. The beneficial influence of the F404Y mutation, established for the Fab-like construct with wild-type C_H_3 domains [16], was also found to benefit analogous constructs with antigen-binding C_H_3 domains, as they caused a positive shift of *T*_*m*_ for about 1 °C (Supplementary Fig. 2). The second transition was recorded at 87.86 ± 0.02 °C for Construct 2, at 83.95 ± 0.045 °C for Construct 3 and 81.59 ± 0.12 °C for Construct 4. Construct 5 was also of high thermostability with two transitions at 71.4 ± 0.02 and 82.63 ± 0.25 °C. Due to the thermostabilizing effect of the F404Y mutation, these were the variants introduced into the full-length IgG-like domain-exchanged antibodies. The mAb^2^ molecule, trastuzumab with the CT6 Fcab replacing the C_H_2–C_H_3 domains, exhibited the transitions at 71.02 ± 0.09, 78.31 ± 0.06 and 80.2 ± 0.015 °C, and differed little from trastuzumab (70.8 ± 0.013, 79.9 ± 0.005 and 81.13 ± 0 °C). Thermal denaturation of Construct 8 was best described with 4 transitions, at 70.8 ± 0.064, 78.8 ± 0.06, 80.8 ± 0.04 and 90.3 ± 0.76 °C, the first corresponding to melting of C_H_2 domains, second and third to melting of the Fab fragments, and the last to melting of heterodimerized C_H_3 domains. The first three melting points were observed to be similar for the analogous bispecific Construct 9 (69.1 ± 0.064, 78.3 ± 0.56, and 79.9 ± 0.13 °C) and the antigen-binding mutations decreased the *T*_*m*_ of heterodimerized C_H_3 domains to 85.53 ± 0 °C. The thermograms of the domain-exchanged antibodies exhibited three transitions, the first one corresponding to the melting of variable domains and C_H_2 domain at 69.4 ± 0.06 °C for Construct 10 and at about 67 °C for Constructs 11–13. The second and third transition, related to denaturation of Fab constant domains and C_H_3 domains, could be observed at 82.14 ± 0.08/90.02 ± 0.18 °C for Construct 10 and 83.14 ± 0.04/87.5 ± 0.06 °C and 77.3 ± 0.6/82.5 ± 0.22 °C for Constructs 11 and 12. The *T*_*m*_s determined for Construct 13 were very similar to those measured for Construct 12 (79.2 ± 0.17 and 82.3 ± 0.67 °C).

**Fig. 3:**
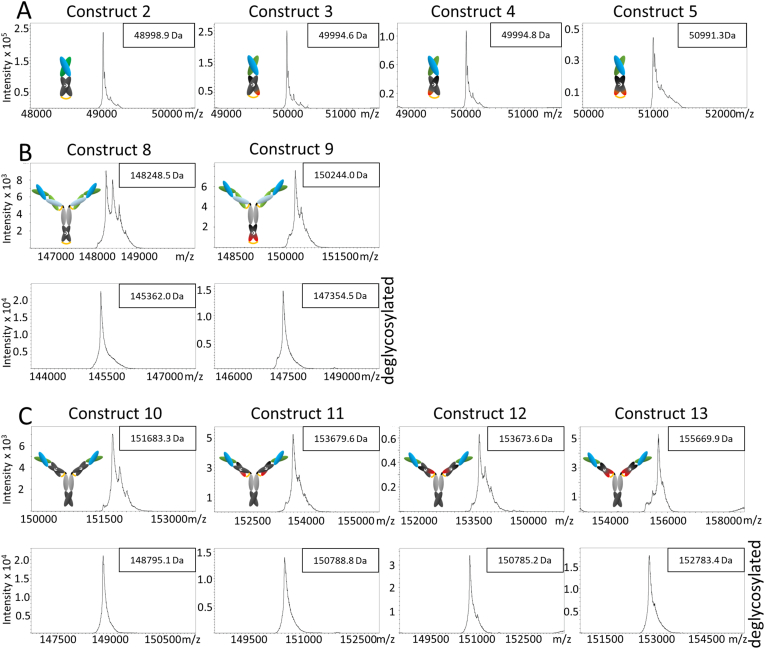
Results of mass spectrometry analysis of (A) Fab-like domain-exchanged constructs; (B) control full-length IgG-like antibodies with heterodimerized C-terminally positioned C_H_3 domains (upper panel: before deglycosylation, lower panel: after deglycosylation); (C) domain-exchanged full-length IgG-like antibodies (upper panel: before deglycosylation, lower panel: after deglycosylation).

To exclude the negative impact of improper heterodimerization on antigen-binding properties of novel bispecific constructs, we examined the composition of bispecific FabCabs and full-length IgG-like bispecific antibodies, and found that correct heterodimer formation could be confirmed for all analyzed molecules (Fig. 3). The molecular weight of monomeric constructs corresponded to the theoretical molecular weight within 0.4 Da (Supplementary Table 3) and the IgG-like constructs diverged by maximally 9.4 Da, and these minimal discrepancies can probably be assigned to different post-translational modifications, such as deamidation, citrulline formation, indole double bond reduction or tryptophan oxidation.

**Fig. 4:**
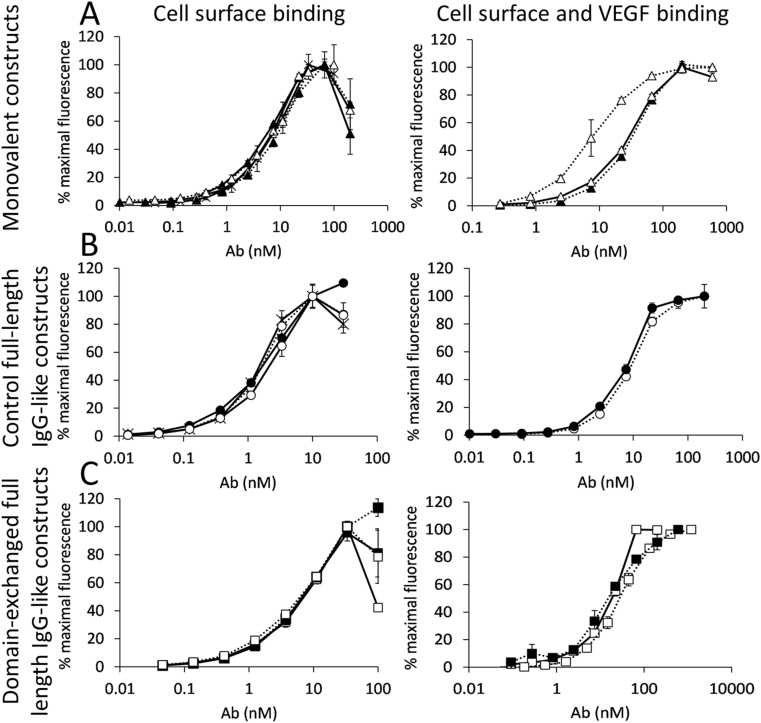
Binding of SK-BR-3 cells, detected once with fluorescent polyclonal antibody conjugate (left panel) and with biotinylated VEGF and fluorescently labelled streptavidin (right panel). (A) Fab and Fab-like constructs: Construct 1 (crosses-full line), Construct 2 (full triangles-full line)_,_ Construct 3 (empty triangles-full line), Construct 4 (full triangles-dotted line) and Construct 5 (empty triangles-dotted line); (B) Control full-length IgG-like constructs with C-terminally positioned C_H_3 domains: Construct 6 (crosses-full line), Construct 7 (full circles-full line), Construct 8 (empty circles-full line) and Construct 9 (empty circles-dotted line); (C) full-length domain-exchanged antibodies: Construct 10 (full squares-full line), Construct 11 (empty squares-full line)_,_ Construct 12 (full squares-dotted line) and Construct 13 (empty squares-dotted line).

Next, antigen-binding properties of the bispecific antibodies were examined. Her2 binding was estimated over the reactivity with the cell-bound antigen and the results are presented in Fig. 4. Fab-like fragments with antigen-binding C_H_3 domains showed the same binding properties as Fab fragment of trastuzumab and the Fab fragments with exchanged wild-type C_H_3 domains (EC_50_ around 7 nM). Domain-exchanged antibodies, on the other hand, stained the antigen-positive cell line about 3-times weaker than TRA and the TRA-CT6 mAb^2^ (EC_50_ of around 5 nM vs. 1.5 nM), a phenomenon also reported for TRA-based domain-exchanged antibody with T366Y/Y407T-heterodimerized wild-type C_H_3 domains replacing the constant domains of the Fab fragment where the binding was 4-fold weaker comparing with TRA [16]. Bispecific binding was evaluated by detection of the SK-BR-3 cell surface bound construct with biotinylated VEGF and streptavidin-Alexa Fluor™ 647. EC_50_ of about 30 nM was found for Constructs 3 and 4 and 8 nM for Construct 5. Constructs 7 and 9 bound at the same level with an EC_50_ of 8 nM. EC_50_ around 20 nM was observed for domain-exchanged bispecific full-length antibodies (Constructs 11–13).

**Fig. 5:**
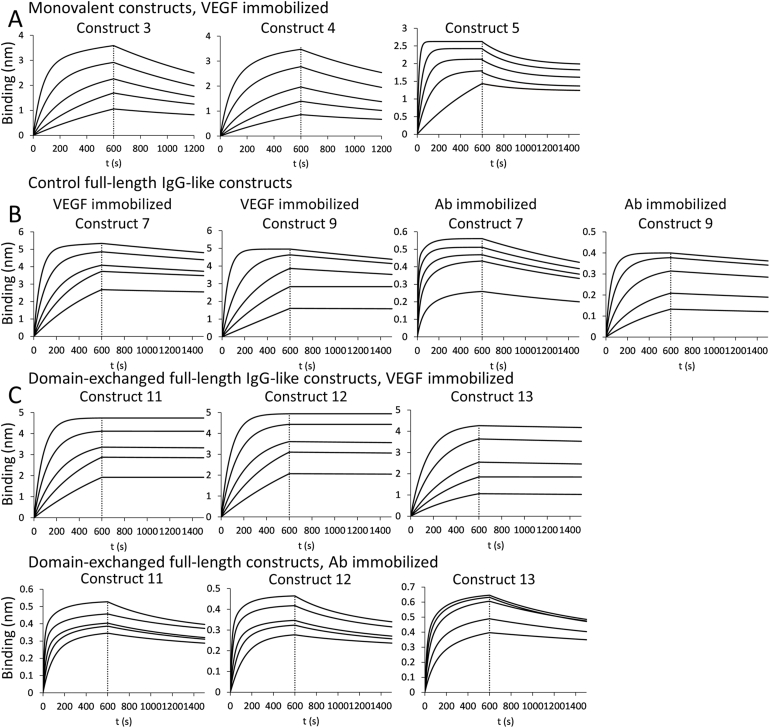
BLI sensorgrams with binding of (A) Fab-like construct 3 (TRA-Fab-C_H_3_ZWHY_/CT6_ZWLY_), 4 (TRA-Fab-CT6_ZWHY_/C_H_3_ZWLY_) and 5 (TRA-Fab-CT6_ZWY_) to VEGF; (B) Construct 7 (TRA-CT6 mAb^2^) and Construct 9 (TRA-CT6_ZW_-FT) to VEGF (left panels) and them capturing VEGF in solution (right panels); (C) full-length domain-exchanged antibody constructs 11 (TRA-C_H_3_ZWHY_/CT6_ZWLY_)_,_ 12 (TRA-CT6_ZWHY_/C_H_3_ZWLY_) and 13 (TRA-CT6_ZWY_) to VEGF (upper panels) and them capturing VEGF in solution (lower panels).

Binding to VEGF via CT6-domains was quantitatively evaluated using BLI and the sensorgrams are presented in Fig. 5. TRA-CT6 mAb^2^ (Construct 7) and its heterodimerized counterpart Construct 9 showed similar binding affinities, 1.67 ± 0.05 nM and 2.08 ± 0.02 nM, and so did the Fab-like Construct 5 for which a binding constant of 2.70 ± 0.06 nM was found. Constructs 6 and 7 exhibited 7.39 ± 1.38 nM and 9.23 ± 0.5 nM binding when the light and heavy chain were fused with an antigen-binding C_H_3 domain, respectively. When such FabCabs were introduced into the whole-length antibody format (Constructs 11 and 12), they could surprisingly show an improved binding to VEGF, with 0.3 ± 0.14 nM and 0.2 ± 0.017 nM for the VEGF-binding domains incorporated into light and heavy chain, respectively. This was due to about 10-fold slower off-rate than could be measured for full-length antibodies with C-terminally positioned VEGF-binding domains. Construct 13 with antigen-binding C_H_3 domains in both heavy and the light chains could bind to VEGF with 0.274 ± 0.002 nM. The parameters of binding kinetics were also evaluated with immobilized full-length IgG-like molecules. While similar values to the previous outlay were obtained for Construct 7 and Construct 9 (2.78 ± 0.003 nM and 1.48 ± 0.02 nM), the domain-exchanged antibodies (Constructs 11, 12 and 13) could capture VEGF with 6.2 ± 0.1 nM, 16.7 ± 0.7 nM and 16.6 ± 0.9 nM affinity.

**Fig. 6:**
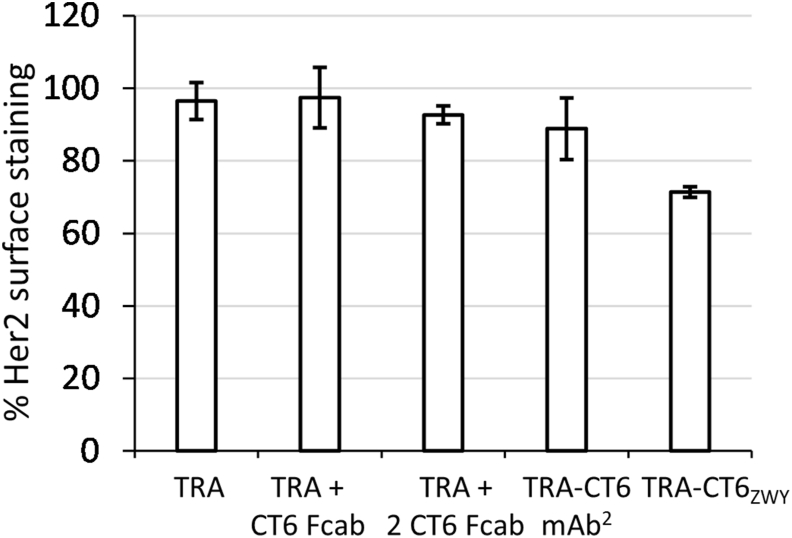
Full-length domain-exchanged antibody TRA-CT6_ZWY_ (Construct 13) reduces surface levels of Her2 in SK-BR-3 cells more potently than TRA, mix of TRA and CT6 Fcab in 1:1 or 1:2 ratio, or TRA-CT6 mAb^2^ (Construct 7).

The ability of the domain-exchanged bispecific antibody to capture 2 VEGF molecules could be harvested for a potent cross-linking of Her2 receptors by this cytokine in a Her2-positive cell line (Fig. 6). For this purpose, we tested the ability of the domain-exchanged bispecific antibody to induce Her2-internalization from the surface of SK-BR-3 cells, which secrete VEGF at a rate of 200 pg/mL/10^6^ cells [24]. Surface levels of Her2 were monitored after incubation with antibodies for 48 h and found to be decreased by 30% upon treatment with the domain-exchanged antibody TRA-CT6_ZWY_ (Construct 13), while they remained within variance of the untreated control with TRA, mixture of TRA and CT6 Fcab in a molar ratio of 1:1 or 1:2, and TRA-CT6 mAb^2^.

## 4 Discussion and conclusions

The colorful collection of bispecific antibody formats available today offers an immense versatility in molecular size, valency, and positioning of the binding sites for different antigens. Among the plethora of linker-assisted, fragment-supported and fusion-based formats, the architectures resembling a native IgG stand out as being preferred for therapeutic use due to a better predictability of expression properties, manufacturability, and lower immunogenicity potential [25]. At the same time, complex constructs relying on co-expression of more than 2 chains in the same cell often require a more laborious optimization of the expression system [4]. Here we attempted to introduce the antigen-binding C_H_3 domains, originating from a symmetric homodimeric IgG-like bispecific mAb^2^, at an unusual position, namely by replacing the constant domains in the Fab fragments to endow such “FabCab” fragment with a second antigen binding specificity. The modified C_H_3-domain of a highly affine VEGF-specific Fcab clone that differs from wild-type C_H_3 in 20 amino acid residues and carries a 5-residue-insertion, could successfully replace the wild-type C_H_3 domains when ZW1-based heterodimerization motif [21] was introduced to support proper pairing between heavy and light chain. Modification of C-terminal residues of the C_H_3 domain to amino acids -GEC to support the formation of the Fab-like C-terminal cysteine bond did not have a negative impact on antigen binding, as has already been observed for another Fcab clone [26].

First, we established that the ZW1-heterodimerization did not impair biophysical properties of the molecule or its antigen-binding characteristics. The TRA-based IgG molecule with the C_H_3 pair with the ZW1-heterodimerization motif (Construct 8) expressed well and exhibited a higher *T*_*m*_ than TRA due to the C-terminally positioned interchain disulfide bond. These favorable properties were also discovered for the bispecific molecule where the C_H_3 domains were replaced with ones carrying mutations relevant to VEGF binding (Construct 9). Importantly, antigen-binding properties of this construct were similar to the mAb^2^ molecule (Construct 7) for antigen specificities mediated both by Fab-arms or variants of C_H_3 domains. This was the validation of molecular design that encouraged the use of heterodimerized C_H_3 domains as antigen-binding moieties within the Fab fragments.

For the construction of the antigen-binding constant domain-exchanged antibody, we first established the need to refrain from the use of simple heterodimerization motifs such as T366Y/Y407T-based “Knobs-into-holes”, as the size analysis in HPLC-SEC revealed an aberrant profile caused by both aggregation and matrix stickiness of the novel molecule. Typically, this mutagenesis method causes a decrease in *T*_*m*_ for about 20 °C [27], while the ZW1-heterodimerization motif used here retained the native thermostability of the heterodimerized C_H_3 domains [21], as we also verified by the comparison of thermal denaturation profiles of TRA (Construct 6) and TRA-C_H_3_ZW_-FT (Construct 8). The antigen-binding mutations caused a decrease in thermostability of the C_H_3 domains of TRA-CT6_ZW_-FT (Construct 9) by about 5 °C, which compares well to the results of comparison of CT6 Fcab and wild-type Fc fragment [15,17]. Domain-exchanged antibodies also exhibited high thermostability, with the first thermal transition still above 67 °C. More prominent destabilization resulted from the exchange of a heavy-variable chain linked antigen-binding constant domain than from the exchange of its light-variable chain linked counterpart, the difference in *T*_*m*_ was 2.5 °C for the monovalent format (Construct 4 vs. Construct 3) and 5 °C for the full-length domain-exchanged antibodies (Construct 12 vs. Construct 11). This phenomenon might be a result of the V_H_-C_H_3_ZWHY_ interface being less optimal than V_L_-C_H_3_ZWLY_, and it will be interesting to discover if this observation extends to other combinations of variable domains and antigen-binding C_H_3 candidates.

High thermostability of all constructs indicated a high degree of order in the molecules, which also contributed to their favorable biophysical properties as they eluted as a single sharp peak in HPLC-SEC at the expected elution volume. All analyzed proteins appeared monodisperse, except for the full-length antibody with antigen-binding C_H_3 domains replacing both constant domains of Fabs TRA-CT6_ZWY_ (Construct 13), which showed about 50% dimeric species that could however be removed using gel filtration. Similar results were obtained also for the equivalent monovalent format TRA-Fab-CT6_ZWY_ (Construct 5).

Previously to determining antigen-binding properties, we established that all studied constructs appear correctly assembled according to mass spectrometry analysis results. The binding to cell-bound Her2 mediated by the variable domains of monovalent formats was indistinguishable from the reactivity of the TRA-Fab-fragment, however there was around 3-fold decrease in the EC_50_ of binding for all domain-exchanged antibodies (Constructs 10–13) comparing with full-length IgGs (Constructs 6–9). In a study that compared cell surface binding of TRA-based domain-exchanged antibody with wild-type C_H_3 domains heterodimerized with T366Y/Y407T motif with TRA, about 4-fold decrease was reported [16]. As the monovalent constructs retained their binding affinity for Her2, this phenomenon can probably be attributed to a different mobility of the Fab arms, previously shown to impact IgG antigen binding and neutralization properties [28]. Interestingly, almost 10-fold stronger binding to the immobilized VEGF comparing with the mAb^2^ molecules was determined, however in the inverse outlay this increase in affinity could not be established. Nevertheless, we attempted to examine the ability of the domain-exchanged antibody to induce Her2 internalization in SK-BR-3 cells, and could determine a more prominent decrease in surface levels of Her2 when cells were treated with TRA-CT6_ZWY_ (Construct 13) than those treated with TRA-CT6 mAb^2^ (Construct 7). SK-BR-3 cells exhibit a very low level of endogenous Her2 internalization [29], but this can be enhanced up to 1000-times upon Her2 oligomer formation caused by Fab-arms of trastuzumab [[30], [31], [32]]. The redirection from recycling to degradation pathway has already been described as a consequence of larger receptor complexes formed upon antibody cross-linking [[33], [34], [35]], which are too large to enter the endocytotic recycling compartment after internalization. Potent Her2 internalization and degradation can lead to receptor depletion and profound “oncogene shock” in oncogene-addicted tumors or to cellular apoptosis [12,36]. On the other hand, such phenomenon could be harvested when the biological activity of the targeting construct is enhanced by its potentiated internalization and prolonged intracellular residence, as was already demonstrated for bi-paratopic Her-2 specific antibody-toxin conjugate that was efficient in *in vivo* eradication of tumors ineligible for anti-Her2 therapy [37].

In conclusion, the novel symmetric bispecific format that features antigen-binding constant domains replacing the Fab constant domains to confer a second specificity to an antibody opens novel possibilities of multiple valency of antigen engagement and also spatial positioning of its binding sites.

## Role of the funding source

The funding source had no influence on study design; on the collection, analysis and interpretation of data; on the writing of the report and on the decision to submit the article for publication. The authors have no conflict of interest to declare.

## Author statement

Filippo Benedetti: Conceptualization, Methodology, Investigation, Data curation, Writing-Original Draft preparation.

Florian Stracke: Conceptualization, Methodology, Investigation, Visualization, Writing-Original Draft preparation.

Katharina Stadlbauer: Methodology, Investigation, Validation, Visualization, Writing-Review and Editing.

Gerhard Stadlmayr: Methodology, Investigation, Validation, Visualization, Writing-Review and Editing.

Florian Rüker: Conceptualization, Supervision, Writing-Review and Editing.

Gordana Wozniak-Knopp: Conceptualization, Methodology, Investigation, Data Curation, Visualization, Supervision, Writing-Original Draft preparation.

## Declaration of competing interest

The authors declare that they have no known competing financial interests or personal relationships that could have appeared to influence the work reported in this paper.
