# Supplementary material for: Bispecific antibodies with Fab-arms featuring exchanged antigen-binding constant domains
Source: Biochem Biophys Rep. 2021 Feb 27;26:100959. doi: 10.1016/j.bbrep.2021.100959 (PMC7920882; doi:10.1016/j.bbrep.2021.100959)
Supplement: Multimedia component 1 [file mmc1.docx]

**Appendix A: Supplementary material**


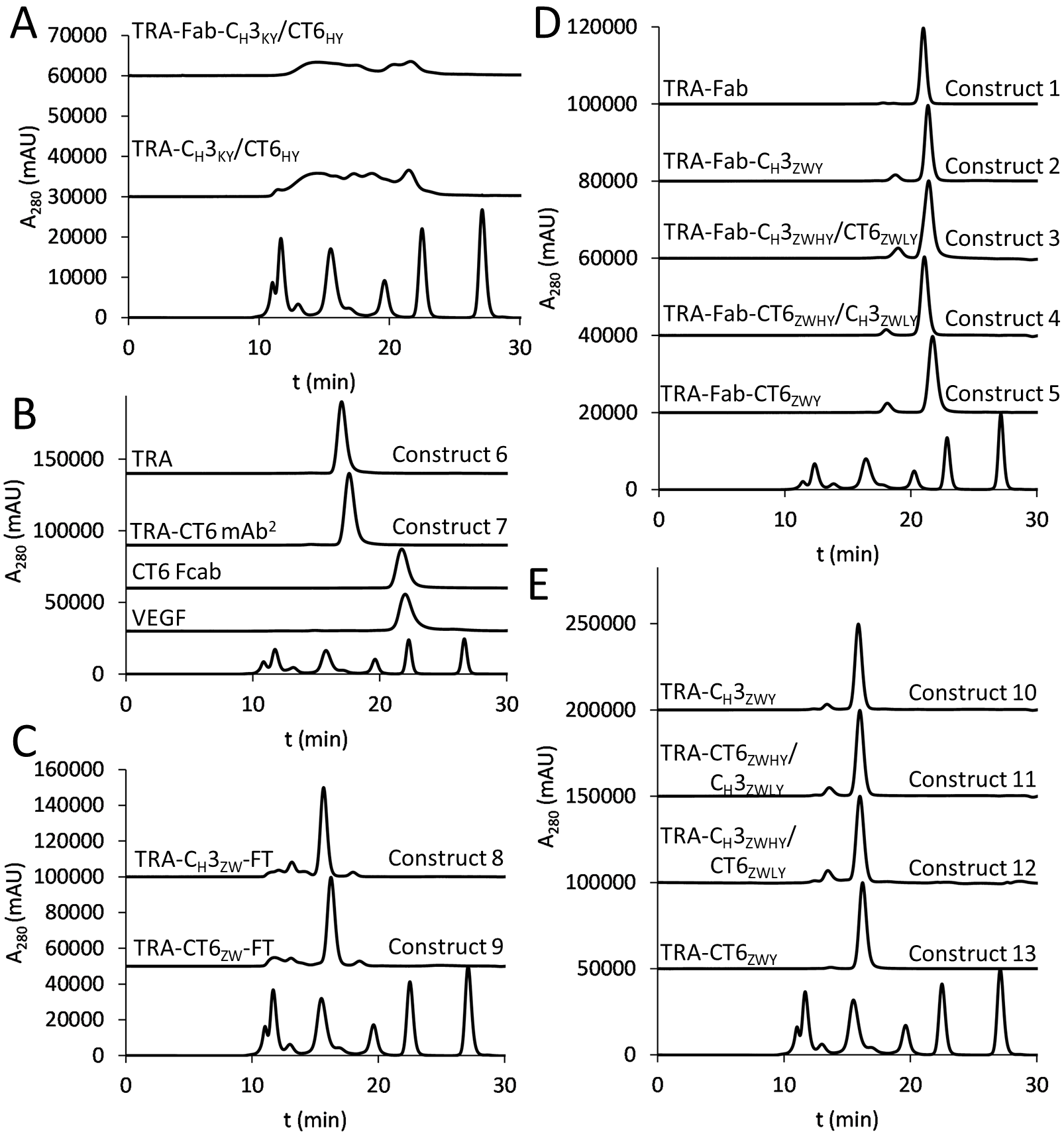


Supplementary Figure 1. SEC-HPLC analysis of (A) T366Y/Y407T heterodimerized Fab-like domain-exchanged construct and domain-exchanged full-length antibody-like construct; (B) control constructs trastuzumab Construct 6), TRA-CT6 mAb^2^ (Construct 7), CT6 Fcab and VEGF antigen; (C) control full-length IgG-like constructs with heterodimerized C-terminal C_H_3-domains TRA-CH3_ZW_-FT (Construct 8) and TRA-CT6_ZW_-FT (Construct 9); (D) TRA-Fab (Construct 1) and domain-exchanged monovalent constructs heterodimerized using ZW1 motif (Constructs 2-5); (E) domain-exchanged full-length IgG-like constructs heterodimerized using ZW1 motif (Constructs 10-13). The molecular weight markers were of 670, 158, 44, 17 and 1.35 kDa in size.


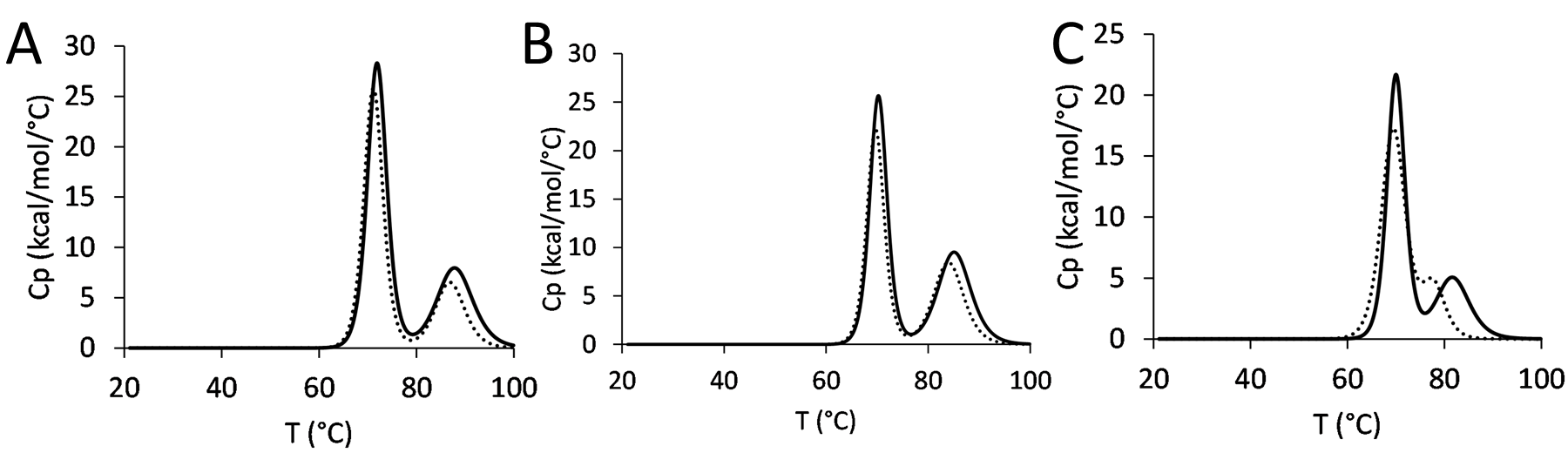


Supplementary Figure 2. DSC analysis of (A) TRA-Fab-C_H_3_ZW_ (dotted line) and TRA-Fab-C_H_3_ZWY_ (full line) (Constructs 2a and 2); (B) TRA-Fab-C_H_3_ZWH_/CT6_ZWL_ (dotted line) and TRA-Fab-C_H_3_ZWHY_/CT6_ZWLY_ (full line) (Constructs 3a and 3); (C) TRA-Fab CT6_ZWH_/C_H_3_ZWL_ (dotted line) and TRA-Fab-CT6_ZWHY_/C_H_3_ZWLY_ (full line) (Constructs 4a and 4).

Supplementary Table 1. Sequences of domain-exchanged antibodies and control constructs. Colored lettering used corresponds to the construct scheme in Figure 1A for the antibody domains: V_L_: green, C_Κ_: light green, V_H_: blue, C_H_1: light blue, C_H_2: gray, C_H_3: black. Heterodimerization mutations are in red. Mutation F404Y is in bold black. The residues mutagenized in CT6-C_H_3 domain are in bold and coloring corresponds to the Figure 1B: blue (AB loop), green (CD loop), yellow (EF loop) and red (C-terminus). Residues GEC intended for the formation of a disulfide bond are underlined with a dotted line.

| Chains of Fab-like constructs |
| --- |
| TRA-V_H_-CH1 |
| EVQLVESGGGLVQPGGSLRLSCAASGFNIKDTYIHWVRQAPGKGLEWVARIYPTNGYTRYADSVKGRFTISADTSKNTAYLQMNSLRAEDTAVYYCSRWGGDGFYAMDYWGQGTLVTVSSASTKGPSVFPLAPSSKSTSGGTAALGCLVKDYFPEPVTVSWNSGALTSGVHTFPAVLQSSGLYSLSSVVTVPSSSLGTQTYICNVNHKPSNTKVDKKVEPKSCDKTHTCPPCPAPELLGGPSVFLFPPKPKDTLMISRTPEVTCVVVDVSHEDPEVKFNWYVDGVEVHNAKTKPREEQYNSTYRVVSVLTVLHQDWLNGKEYKCKVSNKALPAPIEKTISKAKGQPREPQVYTLPPSRDELTKNQVSLTCLVKGFYPSDIAVEWESNGQPENNYKTTPPVLDSDGSFFLYSKLTVDKSRWQQGNVFSCSVMHEALHNHYTQKSLSLSPGK |
| TRA-V_L_-C_Κ_ |
| DIQMTQSPSSLSASVGDRVTITCRASQDVNTAVAWYQQKPGKAPKLLIYSASFLYSGVPSRFSGSRSGTDFTLTISSLQPEDFATYYCQQHYTTPPTFGQGTKVEIKRTVAAPSVFIFPPSDEQLKSGTASVVCLLNNFYPREAKVQWKVDNALQSGNSQESVTEQDSKDSTYSLSSTLTLSKADYEKHKVYACEVTHQGLSSPVTKSFNRGEC |
| TRA-V_H_-C_H_3_KY_ |
| EVQLVESGGGLVQPGGSLRLSCAASGFNIKDTYIHWVRQAPGKGLEWVARIYPTNGYTRYADSVKGRFTISADTSKNTAYLQMNSLRAEDTAVYYCSRWGGDGFYAMDYWGQGTLVTVSSASTKGEPQVYTLPPSRDELTKNQVSLYCLVKGFYPSDIAVEWESNGQPENNYKTTPPVLDSDGS**Y**FLYSKLTVDKSRWQQGNVFSCSVMHEALHNHYTQKSLSLSGEC |
| TRA-V_L_-C_H_3_HY_ |
| DIQMTQSPSSLSASVGDRVTITCRASQDVNTAVAWYQQKPGKAPKLLIYSASFLYSGVPSRFSGSRSGTDFTLTISSLQPEDFATYYCQQHYTTPPTFGQGTKVEIKRTVAEPQVYTLPPSRDELTKNQVSLTCLVKGFYPSDIAVEWESNGQPENNYKTTPPVLDSDGS**Y**FLTSKLTVDKSRWQQGNVFSCSVMHEALHNHYTQKSLSLSGEC |
| TRA-V_H_-CT6_KY_ |
| EVQLVESGGGLVQPGGSLRLSCAASGFNIKDTYIHWVRQAPGKGLEWVARIYPTNGYTRYADSVKGRFTISADTSKNTAYLQMNSLRAEDTAVYYCSRWGGDGFYAMDYWGQGTLVTVSSASTKGEPQVYTLPPSRDEL**RFY**QVSLYCLVKGFYPSDIAVEWESNGQP**DIFPNGL**NYKTTPPVLDSDGS**Y**FLYSKLTV**PYPS**W**LMGTR**FSCSVMHEALHNHYTQK**HLEYQW**GEC |
| TRA-V_L_-CT6_HY_ |
| DIQMTQSPSSLSASVGDRVTITCRASQDVNTAVAWYQQKPGKAPKLLIYSASFLYSGVPSRFSGSRSGTDFTLTISSLQPEDFATYYCQQHYTTPPTFGQGTKVEIKRTVAEPQVYTLPPSRDEL**RFY**QVSLTCLVKGFYPSDIAVEWESNGQP**DIFPNGL**NYKTTPPVLDSDGS**Y**FLTSKLTV**PYPS**W**LMGTR**FSCSVMHEALHNHYTQK**HLEYQW**GEC |
| TRA-V_H_-C_H_3_ZWHY_ |
| EVQLVESGGGLVQPGGSLRLSCAASGFNIKDTYIHWVRQAPGKGLEWVARIYPTNGYTRYADSVKGRFTISADTSKNTAYLQMNSLRAEDTAVYYCSRWGGDGFYAMDYWGQGTLVTVSSASTKGEPQVYVLPPSRDELTKNQVSLLCLVKGFYPSDIAVEWESNGQPENNYLTWPPVLDSDGS**Y**FLYSKLTVDKSRWQQGNVFSCSVMHEALHNHYTQKSLSLSKGEC |
| TRA-V_L_-C_H_3_ZWLY_ |
| DIQMTQSPSSLSASVGDRVTITCRASQDVNTAVAWYQQKPGKAPKLLIYSASFLYSGVPSRFSGSRSGTDFTLTISSLQPEDFATYYCQQHYTTPPTFGQGTKVEIKRTVAEPQVYVYPPSRDELTKNQVSLTCLVKGFYPSDIAVEWESNGQPENNYKTTPPVLDSDGS**Y**ALVSKLTVDKSRWQQGNVFSCSVMHEALHNHYTQKSLSLSGEC |
| TRA-V_H_-CT6_ZWHY_ |
| EVQLVESGGGLVQPGGSLRLSCAASGFNIKDTYIHWVRQAPGKGLEWVARIYPTNGYTRYADSVKGRFTISADTSKNTAYLQMNSLRAEDTAVYYCSRWGGDGFYAMDYWGQGTLVTVSSASTKGEPQVYVLPPSRDEL**RFY**QVSLLCLVKGFYPSDIAVEWESNGQP**DIFPNGL**NYLTWPPVLDSDGS**Y**FLYSKLTV**PYPS**W**LMGTR**FSCSVMHEALHNHYTQK**HLEYQW**GEC |
| TRA-V_L_-CT6_ZWLY_ |
| DIQMTQSPSSLSASVGDRVTITCRASQDVNTAVAWYQQKPGKAPKLLIYSASFLYSGVPSRFSGSRSGTDFTLTISSLQPEDFATYYCQQHYTTPPTFGQGTKVEIKRTVAEPQVYVYPPSRDEL**RFY**QVSLTCLVKGFYPSDIAVEWESNGQP**DIFPNGL**NYKTTPPVLDSDGS**Y**ALVSKLTV**PYPS**W**LMGTR**FSCSVMHEALHNHYTQK**HLEYQW**GEC |
| CT6-Fcab |
| TCPPCPAPELLGGPSVFLFPPKPKDTLMISRTPEVTCVVVDVSHEDPEVKFNWYVDGVEVHNAKTKPREEQYNSTYRVVSVLTVLHQDWLNGKEYKCKVSNKALPAPIEKTISKAKGQPREPQVYTLPPSRDEL**RFY**QVSLTCLVKGFYPSDIAVEWESNGQP**DIFPNGL**NYKTTPPVLDSDGSFFLYSKLTV**PYPS**W**LMGTR**FSCSVMHEALHNHYTQK**HLEYQW**PT |
| Chains of control full-length antibody-like constructs |
| TRA-V_H_-C_H_1-C_H_2-C_H_3 |
| EVQLVESGGGLVQPGGSLRLSCAASGFNIKDTYIHWVRQAPGKGLEWVARIYPTNGYTRYADSVKGRFTISADTSKNTAYLQMNSLRAEDTAVYYCSRWGGDGFYAMDYWGQGTLVTVSSASTKGPSVFPLAPSSKSTSGGTAALGCLVKDYFPEPVTVSWNSGALTSGVHTFPAVLQSSGLYSLSSVVTVPSSSLGTQTYICNVNHKPSNTKVDKKVEPKSCDKTHTCPPCPAPELLGGPSVFLFPPKPKDTLMISRTPEVTCVVVDVSHEDPEVKFNWYVDGVEVHNAKTKPREEQYNSTYRVVSVLTVLHQDWLNGKEYKCKVSNKALPAPIEKTISKAKGQPREPQVYTLPPSRDELTKNQVSLTCLVKGFYPSDIAVEWESNGQPENNYKTTPPVLDSDGSFFLYSKLTVDKSRWQQGNVFSCSVMHEALHNHYTQKSLSLSGEC |
| TRA-V_H_-C_H_1-C_H_2-CT6 |
| EVQLVESGGGLVQPGGSLRLSCAASGFNIKDTYIHWVRQAPGKGLEWVARIYPTNGYTRYADSVKGRFTISADTSKNTAYLQMNSLRAEDTAVYYCSRWGGDGFYAMDYWGQGTLVTVSSASTKGPSVFPLAPSSKSTSGGTAALGCLVKDYFPEPVTVSWNSGALTSGVHTFPAVLQSSGLYSLSSVVTVPSSSLGTQTYICNVNHKPSNTKVDKKVEPKSCDKTHTCPPCPAPELLGGPSVFLFPPKPKDTLMISRTPEVTCVVVDVSHEDPEVKFNWYVDGVEVHNAKTKPREEQYNSTYRVVSVLTVLHQDWLNGKEYKCKVSNKALPAPIEKTISKAKGQPREPQVYTLPPSRDEL**RFY**QVSLTCLVKGFYPSDIAVEWESNGQP**DIFPNGL**NYKTTPPVLDSDGSFFLYSKLTV**PYPS**W**LMGTR**FSCSVMHEALHNHYTQK**HLEYQW**PT |
| TRA-V_H_-C_H_1-C_H_2-C_H_3_ZWH_ |
| EVQLVESGGGLVQPGGSLRLSCAASGFNIKDTYIHWVRQAPGKGLEWVARIYPTNGYTRYADSVKGRFTISADTSKNTAYLQMNSLRAEDTAVYYCSRWGGDGFYAMDYWGQGTLVTVSSASTKGPSVFPLAPSSKSTSGGTAALGCLVKDYFPEPVTVSWNSGALTSGVHTFPAVLQSSGLYSLSSVVTVPSSSLGTQTYICNVNHKPSNTKVDKKVEPKSCDKTHTCPPCPAPELLGGPSVFLFPPKPKDTLMISRTPEVTCVVVDVSHEDPEVKFNWYVDGVEVHNAKTKPREEQYNSTYRVVSVLTVLHQDWLNGKEYKCKVSNKALPAPIEKTISKAKGQPREPQVYVLPPSRDELTKNQVSLLCLVKGFYPSDIAVEWESNGQPENNYLTWPPVLDSDGSFFLYSKLTVDKSRWQQGNVFSCSVMHEALHNHYTQKSLSLSGEC |
| TRA-V_H_-C_H_1-C_H_2-C_H_3_ZWL_ |
| EVQLVESGGGLVQPGGSLRLSCAASGFNIKDTYIHWVRQAPGKGLEWVARIYPTNGYTRYADSVKGRFTISADTSKNTAYLQMNSLRAEDTAVYYCSRWGGDGFYAMDYWGQGTLVTVSSASTKGPSVFPLAPSSKSTSGGTAALGCLVKDYFPEPVTVSWNSGALTSGVHTFPAVLQSSGLYSLSSVVTVPSSSLGTQTYICNVNHKPSNTKVDKKVEPKSCDKTHTCPPCPAPELLGGPSVFLFPPKPKDTLMISRTPEVTCVVVDVSHEDPEVKFNWYVDGVEVHNAKTKPREEQYNSTYRVVSVLTVLHQDWLNGKEYKCKVSNKALPAPIEKTISKAKGQPREPQVYVYPPSRDELTKNQVSLTCLVKGFYPSDIAVEWESNGQPENNYKTTPPVLDSDGSFALVSKLTVDKSRWQQGNVFSCSVMHEALHNHYTQKSLSLSGEC |
| TRA-V_H_-C_H_1-C_H_2-CT6_ZWH_ |
| EVQLVESGGGLVQPGGSLRLSCAASGFNIKDTYIHWVRQAPGKGLEWVARIYPTNGYTRYADSVKGRFTISADTSKNTAYLQMNSLRAEDTAVYYCSRWGGDGFYAMDYWGQGTLVTVSSASTKGPSVFPLAPSSKSTSGGTAALGCLVKDYFPEPVTVSWNSGALTSGVHTFPAVLQSSGLYSLSSVVTVPSSSLGTQTYICNVNHKPSNTKVDKKVEPKSCDKTHTCPPCPAPELLGGPSVFLFPPKPKDTLMISRTPEVTCVVVDVSHEDPEVKFNWYVDGVEVHNAKTKPREEQYNSTYRVVSVLTVLHQDWLNGKEYKCKVSNKALPAPIEKTISKAKGQPREPQVYVLPPSRDEL**RFY**QVSLLCLVKGFYPSDIAVEWESNGQP**DIFPNGL**NYLTWPPVLDSDGSFFLYSKLTV**PYPS**W**LMGTR**FSCSVMHEALHNHYTQK**HLEYQW**GEC |
| TRA-V_H_-C_H_1-C_H_2-CT6_ZWL_ |
| EVQLVESGGGLVQPGGSLRLSCAASGFNIKDTYIHWVRQAPGKGLEWVARIYPTNGYTRYADSVKGRFTISADTSKNTAYLQMNSLRAEDTAVYYCSRWGGDGFYAMDYWGQGTLVTVSSASTKGPSVFPLAPSSKSTSGGTAALGCLVKDYFPEPVTVSWNSGALTSGVHTFPAVLQSSGLYSLSSVVTVPSSSLGTQTYICNVNHKPSNTKVDKKVEPKSCDKTHTCPPCPAPELLGGPSVFLFPPKPKDTLMISRTPEVTCVVVDVSHEDPEVKFNWYVDGVEVHNAKTKPREEQYNSTYRVVSVLTVLHQDWLNGKEYKCKVSNKALPAPIEKTISKAKGQPREPQVYVYPPSRDEL**RFY**QVSLTCLVKGFYPSDIAVEWESNGQP**DIFPNGL**NYKTTPPVLDSDGSFALVSKLTV**PYPS**W**LMGTR**FSCSVMHEALHNHYTQK**HLEYQW**GEC |
| Chains of domain-exchanged full-length antibody-like constructs |
| TRA-V_H_-C_H_3_ZWHY_-C_H_2-C_H_3 |
| EVQLVESGGGLVQPGGSLRLSCAASGFNIKDTYIHWVRQAPGKGLEWVARIYPTNGYTRYADSVKGRFTISADTSKNTAYLQMNSLRAEDTAVYYCSRWGGDGFYAMDYWGQGTLVTVSSASTKGEPQVYVLPPSRDELTKNQVSLLCLVKGFYPSDIAVEWESNGQPENNYLTWPPVLDSDGS**Y**FLYSKLTVDKSRWQQGNVFSCSVMHEALHNHYTQKSLSLSGECDKTHTCPPCPAPELLGGPSVFLFPPKPKDTLMISRTPEVTCVVVDVSHEDPEVKFNWYVDGVEVHNAKTKPREEQYNSTYRVVSVLTVLHQDWLNGKEYKCKVSNKALPAPIEKTISKAKGQPREPQVYTLPPSRDELTKNQVSLTCLVKGFYPSDIAVEWESNGQPENNYKTTPPVLDSDGSFFLYSKLTVDKSRWQQGNVFSCSVMHEALHNHYTQKSLSLSPGK |
| TRA-V_L_-C_H_3_ZWLY_ |
| DIQMTQSPSSLSASVGDRVTITCRASQDVNTAVAWYQQKPGKAPKLLIYSASFLYSGVPSRFSGSRSGTDFTLTISSLQPEDFATYYCQQHYTTPPTFGQGTKVEIKRTVAEPQVYVYPPSRDELTKNQVSLTCLVKGFYPSDIAVEWESNGQPENNYKTTPPVLDSDGS**Y**ALVSKLTVDKSRWQQGNVFSCSVMHEALHNHYTQKSLSLSGEC |
| TRA-V_H_-CT6_ZWHY_-C_H_2-C_H_3 |
| EVQLVESGGGLVQPGGSLRLSCAASGFNIKDTYIHWVRQAPGKGLEWVARIYPTNGYTRYADSVKGRFTISADTSKNTAYLQMNSLRAEDTAVYYCSRWGGDGFYAMDYWGQGTLVTVSSASTKGEPQVYVLPPSRDEL**RFY**QVSLLCLVKGFYPSDIAVEWESNGQP**DIFPNGL**NYLTWPPVLDSDGS**Y**FLYSKLTV**PYPS**W**LMGTR**FSCSVMHEALHNHYTQK**HLEYQW**GECDKTHTCPPCPAPELLGGPSVFLFPPKPKDTLMISRTPEVTCVVVDVSHEDPEVKFNWYVDGVEVHNAKTKPREEQYNSTYRVVSVLTVLHQDWLNGKEYKCKVSNKALPAPIEKTISKAKGQPREPQVYTLPPSRDELTKNQVSLTCLVKGFYPSDIAVEWESNGQPENNYKTTPPVLDSDGSFFLYSKLTVDKSRWQQGNVFSCSVMHEALHNHYTQKSLSLSPGK |
| TRA-V_L_-CT6_ZWLY_ |
| DIQMTQSPSSLSASVGDRVTITCRASQDVNTAVAWYQQKPGKAPKLLIYSASFLYSGVPSRFSGSRSGTDFTLTISSLQPEDFATYYCQQHYTTPPTFGQGTKVEIKRTVAEPQVYVYPPSRDEL**RFY**QVSLTCLVKGFYPSDIAVEWESNGQP**DIFPNGL**NYKTTPPVLDSDGS**Y**ALVSKLTV**PYPS**W**LMGTR**FSCSVMHEALHNHYTQK**HLEYQW**GEC |
| VEGF-antigen |
| VEGF_109_ N75Q |
| GQNHHEVVKFMDVYQRSYCHPIETLVDIFQEYPDEIEYIFKPSCVPLMRCGGCCNDEGLECVPTEESQITMQIMRIKPHQGQHIGEMSFLQHNKCECRPKKD |

Supplementary Table 2. List of constructs and their chain composition.

| No. |  | „heavy“ chain | „light“ chain |
| --- | --- | --- | --- |
|  | **Fab-like constructs** | | |
| 1 | TRA-Fab | TRA-V_H_-C_H_1 | TRA-V_L_-C_Κ_ |
|  | TRA-Fab-C_H_3_KiHY_ | TRA-V_H_-C_H_3_KY_ | TRA-V_L_-C_H_3_HY_ |
|  | TRA-Fab-C_H_3_KY_/CT6_HY_ | TRA-V_H_-C_H_3_KY_ | TRA-V_L_-CT6_HY_ |
|  | TRA-Fab-CT6_KY_/C_H_3_HY_ | TRA-V_H_-CT6_KY_ | TRA-V_L_-C_H_3_HY_ |
|  | TRA-Fab-CT6_KiHY_ | TRA-V_H_-CT6_KY_ | TRA-V_L_-CT6_HY_ |
| 2a | TRA-Fab-C_H_3_ZW_ | TRA-V_H_-C_H_3_ZWH_ | TRA-V_L_-C_H_3_ZWL_ |
| 2 | TRA-Fab-C_H_3_ZWY_ | TRA-V_H_-C_H_3_ZWHY_ | TRA-V_L_-C_H_3_ZWLY_ |
| 3a | TRA-Fab-C_H_3_ZWH_/CT6_ZWL_ | TRA-V_H_-C_H_3_ZWH_ | TRA-V_L_-CT6_ZWL_ |
| 3 | TRA-Fab-C_H_3_ZWHY_/CT6_ZWLY_ | TRA-V_H_-C_H_3_ZWHY_ | TRA-V_L_-CT6_ZWLY_ |
| 4a | TRA-Fab-CT6_ZWH_/C_H_3_ZWL_ | TRA-V_H_-CT6_ZWH_ | TRA-V_L_-C_H_3_ZWL_ |
| 4 | TRA-Fab-CT6_ZWHY_/C_H_3_ZWLY_ | TRA-V_H_-CT6_ZWHY_ | TRA-V_L_-C_H_3_ZWLY_ |
| 5a | TRA-Fab-CT6_ZW_ | TRA-V_H_-CT6_ZWH_ | TRA-V_L_-CT6_ZWL_ |
| 5 | TRA-Fab-CT6_ZWY_ | TRA-V_H_-CT6_ZWHY_ | TRA-V_L_-CT6_ZWLY_ |
|  | **Control full-length IgG-like constructs** | | |
| 6 | TRA | TRA-V_H_-C_H_1-C_H_2-C_H_3 | TRA-V_L_-C_Κ_ |
| 7 | TRA-CT6 mAb^2^ | TRA-V_H_-C_H_1-C_H_2-CT6 | TRA-V_L_-C_Κ_ |
| 8 | TRA-C_H_3_ZW_-FT | TRA-V_H_-C_H_1-C_H_2-C_H_3_ZWH_ and TRA-V_H_-C_H_1-C_H_2-C_H_3_ZWL_ | TRA-V_L_-C_Κ_ |
| 9 | TRA-CT6_ZW_-FT | TRA-V_H_-C_H_1-C_H_2-CT6_ZWH_ and TRA-V_H_-C_H_1-C_H_2-CT6_ZWL_ | TRA-V_L_-C_Κ_ |
|  | **Domain-exchanged full-length IgG-like constructs** | | |
|  | TRA-C_H_3_KY_/CT6_HY_ | TRA-V_H_-C_H_3_ZWKY_-C_H_2-C_H_3 | TRA-V_L_-CT6_HY_ |
| 10 | TRA-C_H_3_ZWY_ | TRA-V_H_-C_H_3_ZWHY_-C_H_2-C_H_3 | TRA-V_L_-C_H_3_ZWLY_ |
| 11 | TRA-CT6_ZWHY_/C_H_3_ZWLY_ | TRA-V_H_-CT6_ZWHY_-C_H_2-C_H_3 | TRA-V_L_-C_H_3_ZWLY_ |
| 12 | TRA-C_H_3_ZWHY_/CT6_ZWLY_ | TRA-V_H_-C_H_3_ZWHY_-C_H_2-C_H_3 | TRA-V_L_-CT6_ZWLY_ |
| 13 | TRA-CT6_ZWY_ | TRA-V_H_-CT6_ZWHY_-C_H_2-C_H_3 | TRA-V_L_-CT6_ZWLY_ |

Supplementary Table 3. Molecular weight of novel constructs, calculated and determined with mass spectrometry before and after deglycosylation.

| No. |  | Calculated MW | Measured MW |  |  |
| --- | --- | --- | --- | --- | --- |
|  | **Monovalent constructs** | (Da) | (Da) |  |  |
| 2 | TRA-Fab-C_H_3_ZWY_ | 48998.59 | 48998.9 |  |  |
| 3 | TRA-Fab-C_H_3_ZWHY_/CT6_ZWLY_ | 49994.87 | 49994.6 |  |  |
| 4 | TRA-Fab-CT6_ZWHY_/C_H_3_ZWLY_ | 49994.87 | 49994.8 |  |  |
| 5 | TRA-Fab-CT6_ZWY_ | 50991.15 | 50991.3 |  |  |
|  |  | Deglycosylated | | Glycosylated | |
|  |  | Calculated MW | Measured MW | Calculated MW | Measured MW |
|  |  | (Da) | (Da) | (Da) | (Da) |
|  | **Control full-length IgG-like constructs** | | | | |
| 8 | TRA-C_H_3_ZW_-FT | 145361.07 | 145362.0 | 148248.17 | 148248.5 |
| 9 | TRA-CT6_ZW_-FT | 147353.64 | 147354.5 | 150240.74 | 150244.0 |
|  | **Domain-exchanged full-length IgG-like constructs** | | |  |  |
| 10 | TRA-C_H_3_ZWY_ | 148790.62 | 148795.1 | 151677.72 | 151683.3 |
| 11 | TRA-CT6_ZWHY_/C_H_3_ZWLY_ | 150783.18 | 150785.2 | 153670.28 | 153673.6 |
| 12 | TRA-C_H_3_ZWHY_/CT6_ZWLY_ | 150783.18 | 150788.8 | 153670.28 | 153679.6 |
| 13 | TRA-CT6_ZWY_ | 152775.74 | 152783.4 | 155662.84 | 155669.9 |
